# Congenital Defects in Actin Dynamics of Germinal Center B Cells

**DOI:** 10.3389/fimmu.2019.00296

**Published:** 2019-03-06

**Authors:** Minghui He, Lisa S. Westerberg

**Affiliations:** Department of Microbiology Tumor and Cell Biology, Karolinska Institutet, Stockholm, Sweden

**Keywords:** germinal center, B cell receptor, immune synapse, actin cytoskeleton, antibodies

## Abstract

The germinal center (GC) is a transient anatomical structure formed during the adaptive immune response that leads to antibody affinity maturation and serological memory. Recent works using two-photon microscopy reveals that the GC is a highly dynamic structure and GC B cells are highly motile. An efficient selection of high affinity B cells clones within the GC crucially relies on the interplay of proliferation, genome editing, cell-cell interaction, and migration. All these processes require actin cytoskeleton rearrangement to be well-coordinated. Dysregulated actin dynamics may impede on multiple stages during B cell affinity maturation, which could lead to aberrant GC response and result in autoimmunity and B cell malignancy. This review mainly focuses on the recent works that investigate the role of actin regulators during the GC response.

The germinal center (GC) is the site where B cells can modify their B cell receptor (BCR) affinity for antigen by expression of activation induced deaminase (AID), proliferation, and selection. The outcome will be plasma cells and memory B cells that have acquired B cell receptors (BCR) with higher affinity for antigen. During the last 10 years, the dynamics of GC B cells have been investigated by usage of intravital two photon microscopy and revealed an enormous dynamics of GC B cells in migration pattern and interactions with follicular dendritic cells (FDCs) and T follicular helper (Tfh) cells (1–3). A long-standing question about how the antigen is delivered to the FDC network has also been revealed. Small antigens can diffuse into the FDC network by the conduit system (4). Migratory B cells in the marginal zone (MZ) of the spleen and B cells close to the sinusoid macrophages in lymph nodes (LN) can capture antigen by the B cell complement receptors such as CD21 and deliver the antigen into the FDC network (5).

The GC reaction relies on the interplay between cell migration, cell-cell interaction, and cell proliferation. The GC is anatomically divided into the dark zone (DZ) and light zone (LZ). The DZ is the site where B cells have high expression of AID that induces somatic hypermutation (SHM) and Ig class switch recombination (Ig CSR) in the genes encoding the Ig heavy and light chains. The LZ is the site for B cell competition and selection to obtain B cells with highest affinity for antigen. Recent migratory B cell from the DZ compete for retrieval of native antigen on follicular dendritic cells (FDCs). BCR binding of antigen leads to endocytosis and processing of antigen for loading on MHC class II molecules (6–9). This process relies on that B cells form two types of immunological synapses, the first synapse will polarize the machinery for BCR endocytosis for antigen retrieval from FDCs and the second synapse is formed by MHC class II—peptide interaction with T cell receptors (TCR)s on Tfh cells (8). During extraction of antigen from the immune synapse by B cells, the strength and timing of mechanical forces in immune synapses can promote affinity discrimination (10, 11). The antigen presenting B cells interact with Tfh cells that provide co-stimulation and cytokines such as IL-21 and IL-4. The B cell expressing a BCR that have acquired highest affinity for the antigen will acquire more antigen for MHC class II presentation and outcompete B cells expressing a BCR with lower affinity for antigen (3). An estimated 10% of the B cells migrate back to the DZ (3, 12) to undergo more SHM to increase the BCR affinity for antigen. The B cells that have acquired higher affinity for antigen can undergo differentiation to plasma cells and memory cells (13). Whereas the differentiation program to become a plasma cell is defined in quite detail, the memory B cell differentiation program has only recently started to be identified. It is clear that the cell fate decisions that B cells make in the GC are well characterized and coordinated by expression of transcription factors. Pax5 is critical to maintain the GC B cell phenotype. Increased expression of IRF4 and downregulation of Pax5 is the first differentiation step toward plasmablasts and followed by upregulated expression of Blimp1 and Xbp1 in fully differentiated plasma cells. This induces a loss of B cell identity and plasma blasts leave the GC to migrate to the B-T cell bridging areas. The GC response is orchestrated by coordinated changes in cell shape to migrate between the DZ and LZ and to communicate with FDCs and Tfh cells in the LZ.

During the process of finding interaction partners, GC B cells rapidly change cell shape and polarization by forming leading edge protrusions and trailing uropods (14). It is therefore not surprising that inborn errors in genes that regulate the actin cytoskeleton lead to aberrant GC formation. What is perhaps more surprising is that specific mutations lead to development of autoreactive GCs, suggesting that the effects on discriminating the self and non-self B cell clones during the GC reaction is skewed. The importance of actin dynamics and generation of force in the B cell immune synapse has recently been described (11, 15). Investigation of patients with primary immunodeficiency diseases due to inborn errors in B cell responses provides important information about B cell dysfunction in severe disease (16). To understand aberrations in the GC reaction, animal models provide in depth analysis of the anatomical structure in secondary lymphoid organs and the outcome measured as plasma cell generation and antibody production (Figure 1). Here we review recent progress in understanding how cytoskeletal regulators leading to Arp2/3 mediated actin polymerization regulate the B cell fate during the GC response (Table 1). This axis of regulation to actin dynamics involves B cell receptor (BCR) signaling to guanine exchange factors (GEFs) that activate the small GTPases of the Ras homology (Rho) family. Rho GTPases binds to and activates the Wiskott-Aldrich syndrome (WASp) family proteins for actin polymerization by the Arp2/3 complex.

**Figure 1:**
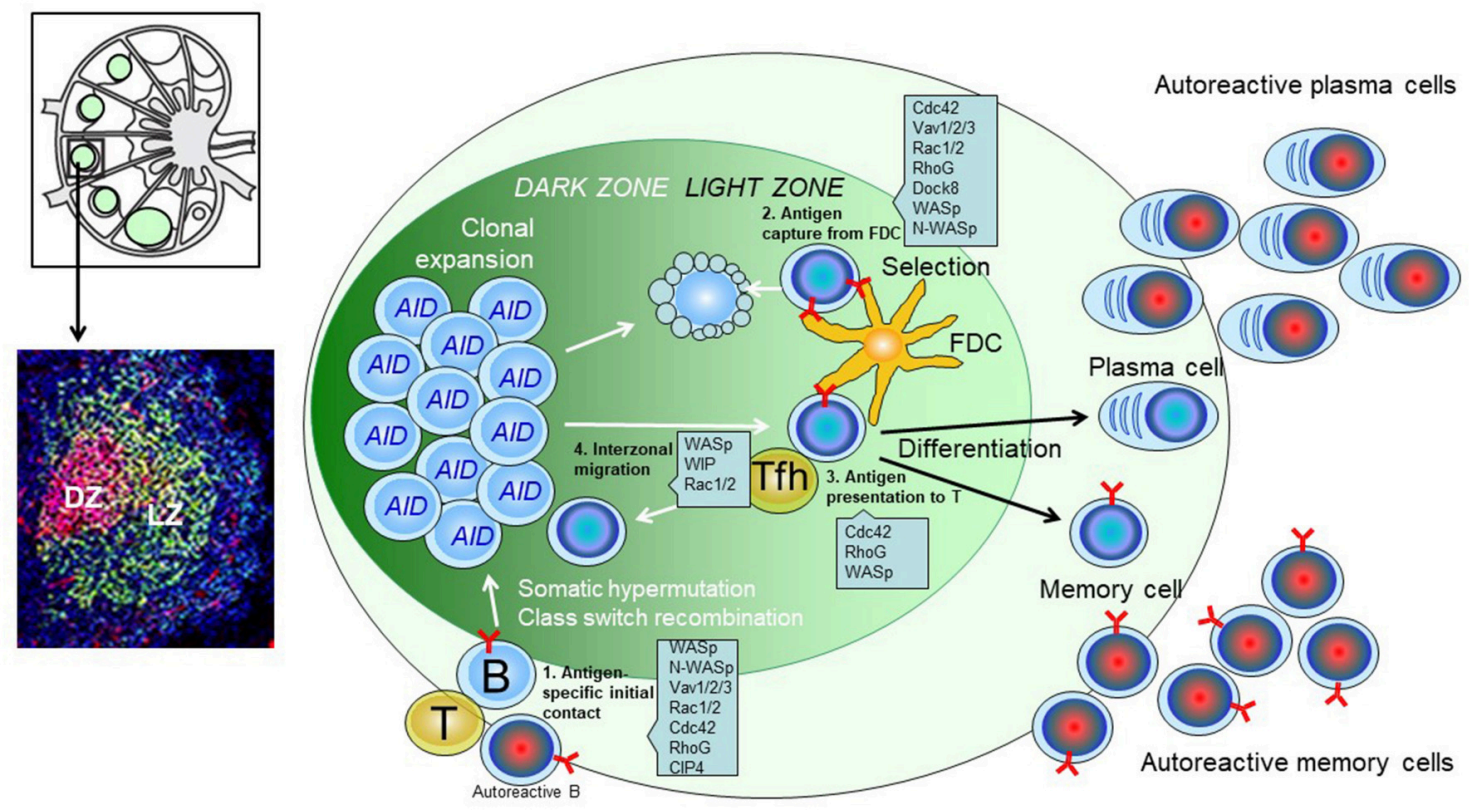
Predicted role of actin regulators during the GC response of B cells. **(Upper left)**: Schematics of the lymph node structure. **(Lower left)**: Immunohistochemistry of a single GC. Red: GL7; Green: CD21/35; Blue: B220. **(Right)**: Schematic of a GC. Antigen-engaged B cells meet antigen experienced Tfh cells and enter the B cell follicle to initiate a GC response. B cells in dark zone express AID (activation-induced cytidine deaminase) and undergo somatic hypermutation. B cell clones that successfully mutate the BCR migrate to light zone, capture antigens deposit on the surface of FDC (follicular dendric cells), and present the antigen to Tfh (T follicular helper) cells. The positive selected B cell clones can differentiate into plasma cells, memory cells or migrate back to dark zone for further mutations and selection.

**Table 1:** B cell development and periphery B cell response in actin regulator deficiency.

	**Vav1^−/−^**	**Vav2^−/−^**	**Vav1^−/−^ Vav2^−/−^ (Vav1+2+3 triple KO)**	**Dock8^−/−^**	**Rac1^−/−^**	**Rac2^−/−^**	**Rac1^B^Rac2^−/−^**	**Cdc42^−/−^**	**WASP^−/−^**	**WIP^−/−^**	**WASP^−/−^ N-WASP^−^/^−^**	**CIP4**	**RhoG**	**Dock10**	**Dock11**
Pro-B/pre-B	→	→	→	→	→	→	→	6 → ↓	→	→		→			FrA↑
Immature B	→	→	3 → ↑	→	→	→	→	→	→	→		→			→
Transitional 1	→	→	↓		→	↓	↓* ↓*	↓	→		→			→	
Transitional 2	↑	→	↓		→	↓	↓ ↓	↓	→		→			→	
Marginal zone B	→		↓	↓	→	↓	↓ ↓	↓	↓		↓			→	↓
Follicular B	→	↓	↓	→	→	Slight ↓	↓↓	↓	→	→	↓			→	→
B1 B	↓	→	↓↓	↓	→	Slight ↓	↓↓	↓	→	→	a → b ↓			→	→
T-dependent	Vsv; Np-ova	DNP-KLH				TNP-SRBC	TNP-SRBC	TNP-SRBC	TNP-KLH	TNP-KLH; Virus	TNP-KLH; NP-KLH; apop cells	KLH	NIP-OVA	TNP-SRBC	NP-CGG
Germinal center	1 → ↓	↓		↓		→	→	↓	↑	↓	9↓↑	↓	10 → ↓	→	
Dark zone/Light zone						→	→				Apop imm↓			→	
Plasma cell				↓		→	→	↓	↑	↓	→			→	
IgM	Serum ↓	Serum →	Serum ↓↓			→	→	Serum ↓ ↓		8 → ↓	↑	Serum →	Serum →	→	→
IgG	serum ↓	Serum ↓	Serum ↓↓			→	→	Serum ↓↓	→		↓	Serum ↓	Serum ↓	→	→
T-independent	TNP-Ficoll	DNP-Ficoll	TNP-Ficoll			TNP-LPS	TNP-LPS	TNP-Ficoll	PPSV;	TNP-LPS; TNP-Ficoll	TNP-Ficoll	TNP-Ficoll/LPS			TNP-Ficoll
IgM	→	↓	↓↓			↓	↓↓	↓	MZB ↓		↓	→			→
IgG2b	IgG →		↓↓			↑	↓			IgG →		→			
IgG3	↓	↓	↓↓			→	↓	↓			↓	→			→
In vitro anti-BCR response															
Cell proliferation	↓	2 → ↓	↓↓	→		↓	↓↓	↓	↑	↑		→		→	
Ca^2+^ flux	↓	↓	↓↓	→		↓		↓	↑	↑					
IS formation	↓	↓		↓	→	↓									
In vitro LPS response				**To CpG response**											
Proliferation	→	→	↓	↓		4 → ↓	5 → ↓	7 → ↓	↑	↑		→		↓	→
IgM				↓				↓						→	
IgG2b				↓		↑	↑↑		↑					CSR →	
In vitro anti-CD40+IL4 response															
Proliferation	→	→	→	→		↓	↓↓	→	↑	↑		→		↓	
IgG1				→		→	→	CSR →	↑	↑		→		CSR →	
Chemotaxis															
CXCL12				→		↓		→	↓	↓	↓		→	→	→
CXCL13				→				→	↓	↓			→	→	→

*Summary of previous studies investigated the role of actin regulators in different aspects of B cells response by using transgenic mouse models or patients periphery blood mononuclear cells. → Normal, ↓ reduced, ↑ increased compare to control. BCR, B cell receptor; IS, immune synapse; CSR, class switch recombination; SRBC, sheep red blood cells. VSV, Vesicular stomatitis virus*.

*1. VSV, GC normal; NP-OVA, GC reduced. 2–9. Different laboratory comes to different conclusions, depend on experimental model, immunogen and methods used. 10. To soluble Ag, GC normal; to particle Ag, GC reduced*.

## GEFs: Dock Family and Vav1-3

GEFs activate small GTPases by stimulating the exchange of guanosine diphosphate (GDP) to guanosine triphosphate (GTP). GTPase activating proteins (GAPs) stimulate GTP hydrolysis thereby reinstating the GDP-bound form of the GTPases to terminate their signaling. Regulated by GEFs and GAPs, the Rho family GTPases cycle between a GDP-bound inactive form and a GTP-bound active form (17). The GEFs that regulate Rho GTPases called RhoGEFs fall into two different classes: the dedicator of cytokinesis (Dock) family of proteins including Dock 1–11 and the diffuse B-cell lymphoma (Dbl) family including Vav1-3.

### Dock Family Proteins

Dock8, a GEF for Rho GTPases, was first identified in a yeast two hybrid screening for Cdc42 binding partners (18). Dock8 deficiency in patients leads to multiple immune disorders including T and B cell deficiency, increased cutaneous viral infection, severe atopy with elevated serum IgE level, and compromised antibody response (19, 20). Detailed analysis of patient PBMCs reveal a reduced proportion of T cells and slightly elevated CD19^+^ B cells. However, among the periphery blood B cells in Dock8 deficient patients, there is almost a complete lack of CD27^+^ B cells including switched memory (IgD^−^CD27^+^) B cells and non-switched memory (MZ like, IgD^+^CD27^+^) B cells. This is associated with reduced serum IgG and IgM response to vaccination and lack of serological memory in the patients (21). These data suggests that Dock8 deficiency leads to a compromised GC response. In depth studies of a Dock8^−/−^ mice shows reduced naïve T cells, MZ B, and B1 B cell subsets. Upon antigen challenge, the Dock8^−/−^ B cell response in the extrafollicular pathway is comparable to that of wildtype B cells. However, the GC response and antibody affinity maturation of Dock8^−/−^ B cells is greatly compromised although the rate of SHM is comparable to wildtype cells. The reduced GC response is probably not due to compromised entry of Dock8^−/−^ B cells into the GCs. Because during the early GC response (day 2–5), Dock8^−/−^ and wildtype B cells occupy the GC area equally well. However, at the later time points, Dock8^−/−^ GC B cells gradually lose the competition, suggesting a critical role of Dock8 for GC B cell persistence or survival. This defect may be caused by the compromised immune synapse formed during the selection stage of GC B cells in the LZ, which may provide crucial survival signal to the GC B cells (22).

### Dock2

Dock2 is predominantly expressed in hematopoietic cells and human loss-of-function mutations result in early onset of invasive bacterial and viral infection, T cell lymphopenia, and decreased antibody responses (23). Detailed analysis of Dock2-deficient patient cells reveal defective T cell and B cell responses upon antigen stimulation as a result of impaired Rac activation and actin polymerization. Analysis of B cell specific Dock2^−/−^ (CD19-Cre x Dock2^fl/fl^) mice and cell lines have identified a critical role of Dock2 in B cells during the antigen induced immune synapse formation, cell proliferation, and plasma cell differentiation (24, 25). CD19-Cre x Dock2^fl/fl^ mice have normal B cell development in bone marrow from the pro-/pre-B cell stage to the immature B cell stage. However, there is a dramatic decrease in the mature B cell subsets including transitional B cell, marginal zone B, and follicular B cells (25, 26). This could at least partly result from compromised cell migration to chemokines of Dock2^−/−^ B cells (26, 27). CD19-Cre x Dock2^fl/fl^ mice have decreased IgG1 and IgG2b antibody response to T cell dependent (TD) antigen. Examination of the GC response show that Dock2 deficiency does not affect GC B cell formation and Ig class switching, whereas the GC B cell proliferation and differentiation into plasma cells are greatly compromised (25). This could be caused by a defective immune synapse formation at the selection stage in the LZ and therefore lack of survival and differentiation signal from the Tfh cells.

### Dock10

Other proteins in the Dock family have been associated with B cell biology and the GC response. In a screen for genes upregulated by IL-4 activation of B cells, Dock10 was one of the highest expressed genes (28, 29). Dock10^−/−^ mice have reduced numbers of B cells in secondary lymphoid organs, and FO B cells display elevated expression of membrane CD23 (30). These results suggest that Dock10 plays a role in B-cell lymphopoiesis in secondary lymphoid tissue. However, specific deletion of Dock10 in B cells was associated with a mild phenotype with normal B cell development and normal B cell spreading, polarization, motility, chemotaxis, aggregation, and Ig class switching. Dock10^B^ B cells showed lower proliferation in response to anti-CD40 and IL-4 stimulation *in vitro* and Dock10^B^ mice had reduced IgG response to NP-KLH *in vivo* (28). This suggest that IL-4 induced activation of B cells was decreased both *in vitro* and *in vivo* but that most B cell responses were functional in the absence of Dock10, rising the interesting question if the closest homologs to Dock10, Dock 9, and Dock11 may have redundant activity in B cells.

Dock11 is highly expressed in lymphocytes and Dock11-deficient mice have reduced development of splenic MZ B cells (31). Dock11^−/−^ mice show a normal antibody response to T cell independent (TI) antigens and TD antigens, TNP-LPS, TNP-Ficoll, and NP-CGG (32). This indicates that Dock11^−/−^ mice have a normal GC response although generation of high affinity antibodies was not examined in detail.

### Vav1, Vav2, and Vav3

Vav proteins were first described as proto-oncogenes acting as substrates for tyrosine protein kinase activity (33). Recent studies examining the role of Vav family proteins, including Vav1, Vav2, and Vav3, in lymphocytes have revealed their critical function to link lymphocyte antigen receptor activation to actin cytoskeleton dynamics. Vav1, Vav2, and Vav3 share more than 50% homology in the protein sequences, all of which are composed of a Dbl-homologous (DH) domain, pleckstrin homology (PH) domain, SH2/SH3 domain, proline rich area, and a calponin homology (CH) domain (34). Reduced Vav1 expression has been detected in common variable immunodeficiency (CVID) patients with defective TCR mediated signaling (35). Vav1 expression is mainly restricted to the haematopoietic lineage cells (36). Although Vav1 has been shown to play a critical role in T cell development and activation by regulation of TCR signaling, B cell development of Vav1^−/−^ mice seems largely unaltered, except a profound reduction of B1 B cells in the peritoneal cavity (37–39). The *in vivo* response of Vav1^−/−^ B cells to T-independent antigens (both TI-1 and TI-2) is comparable to wildtype cells as measured by production of antigen specific IgM. However, despite normal formation of GCs in response to vesicular stomatitis virus (VSV), antigen specific IgG responses are reduced. In response to NIP-OVA, Vav1^−/−^ mice completely lack GCs, which probably leads to reduced antigen specific IgG1 and IgG2b. Vav1 is highly expressed in all haematopoietic cells, whereas Vav2 shows the highest expression in splenic mature B cells when compared to other B cell subsets, suggesting an important role of Vav2 in mature B cell homeostasis. Consistently, Vav2^−/−^ mice seem to have a development block from the immature/transitional B cell stage to the mature B cell stage. There is also reduced response to both TI and TD antigens of Vav2^−/−^ B cells when compared to wildtype cells. In response to TNP-KLH, Vav2^−/−^ mice show an 80% reduction in the GC B cells. Because T cell subsets and function are suggested to be unaltered in Vav2^−/−^ mice, it is likely that the compromised GC response in Vav2^−/−^ mice results from a B cell intrinsic defect (40, 41).

All three proteins of Vav1, Vav2, and Vav3 are quickly phosphorylated after the antigen receptor engagement. Since previous data demonstrates relative mild defect in Vav1^−/−^ and Vav2^−/−^ single knockout mice, Vav1, Vav2, and Vav3 may have functional redundancy downstream of BCR activation. The collected experimental data so far supports this hypothesis. Vav1^−/−^ Vav2^−/−^ double knockout mice and Vav1^−/−^ Vav2^−/−^ Vav3^−/−^ triple knockout mice have a more severe B cell deficiency, including a developmental block at the immature/transitional B cell stage in bone marrow and spleen, reduced serum level of IgM and IgG, defective response to TI and TD antigens and greatly compromised cell proliferation and calcium flux upon BCR stimulation (42).

## Small Rho GTPases

The Rho family belongs to the Ras super family of small GTPases and like other Ras-related proteins, most of the Rho GTPases adopt either active GTP-bound or inactive GDP-bound conformational states. The important role of the small Rho GTPases in regulation of actin dynamics was first characterized by Alan Hall and coworkers that showed induction of specific actin structures when microinjected into fibroblasts (43–46). Cell division control protein 42 homolog (Cdc42), Ras-related C3 botulinum toxin substrate 1 (Rac1), and Ras homolog gene family, member A (RhoA) has been the prototypic members of the family of small Rho GTPases. Cdc42 microinjection into fibroblasts induces membrane filopodia and Cdc42 regulates cell polarity and cell division (44). Rac1 induces membrane ruffles and lamellipodia and RhoA regulates stress fiber formation (46, 47). It was later shown that such actin dependent structures is induced by Cdc42, Rac1, and RhoA in other cell types including B cells (48, 49). Studies from many laboratories have revealed extensive cross-talk among the Rho GTPases, not the least in hematopoietic cells that express many variants of the Rho GTPases (50).

### Cdc42

The small GTPase Cdc42 can mediate the interaction between actin and microtubules and regulate cell shape and polarity. Cdc42 coordinates actin polymerization by direct binding to WASp and N-WASp (51–53) and coordinates the microtubule cytoskeleton by binding to the Cdc42 interacting protein (CIP4) that directly regulates microtubule assembly (54, 55). *In vitro*, dominant negative mutants of Cdc42 interfere with B cell formation of cytoskeletal responses such as formation of filopodia, and cell polarization and migration (48, 49). Two patients with unrelated Cdc42 mutations have been reported recently (56, 57). The patients are characterized with developmental delay, macro thrombocytopenia, and lymphedema. Repeated upper respiratory infection and chronic leukocytopenia has been observed in one of the patients, indicating a mild form of immunodeficiency. Using animal models, Cdc42 has a non-redundant role during B cell development since deletion in early B cell progenitors results in a severe reduction in the numbers of mature B cells (58, 59). Using CD19-Cre for deletion of a floxed Cdc42 allele, Cdc42-deficient B cells have decreased phosphorylation of Akt upon BCR activation and reduced BAFFR signaling leading to reduced proliferation and increased apoptosis (58). Mice with B cell-specific deletion of Cdc42 induced a reduced antibody response to TNP-Ficoll and NP-KLH. Early deletion of Cdc42 during B cell development using mb1-Cre x Cdc42^flox/flox^ mice, led to reduced B cell number in spleen and LN and antibody titers reaching the detection limit (59). This led to abolished capacity to generate a high affinity antibody response to NP-KLH and reduced GC response to Influenza A virus. Together this suggests that Cdc42 serves an important role during B cell development in the bone marrow. Using the super resolution microscopy technique dSTORM, Cdc42 KO B cells showed increased dispersion of IgM nanoclusters and decreased BCR induced signaling leading to reduced internalization of antigen (59). Using two-photon microscopy, Cdc42 KO B cells formed fewer contacts with antigen-specific T cells (59).

Cdc42^−/−^ B cells migrate normally to chemokines *in vitro* (58, 60), but have reduced capacity to home to the B cell follicles in the spleen (60). To exclude the effect of Cdc42 deletion on B cell development and the effect of Cdc42 deficiency on positioning in LNs and splenic white pulp, inducible deletion of Cdc42 by crossing Cdc42^flox/flox^ mice with mb1-Cre-ERT2 mice was employed (60). This approach allowed for specific deletion of Cdc42 in B cells that had already entered the B cell follicles. Inducible deletion of Cdc42 in B cells led to reduced number of splenic MZ B cells and follicular B cells. Upon antigen challenge with the particulate antigen sheep red blood cells (SRBC), Cdc42^B−ERT2^ had reduced formation of GCs. In response to NP-KLH, Cdc42^B−ERT2^ B cells showed reduced capacity to induce NP-specific antibodies. This was associated with reduced capacity to present antigenic peptides to T cells *in vitro* (60). Moreover, Cdc42^B−ERT2^ B cells failed to form membrane extensions rich in tubulin and formed only short membrane protrusions that do not contain tubulin.

Together, these studies suggest that Cdc42 plays a role both during B cell development and in GC response and Cdc42 deficient B cells fail to regulate formation of membrane extensions and to interact with T follicular helper cells.

### Rac1 and Rac2

The Rac proteins were first identified in Snyderman's laboratory in 1989 (61). Sequence analysis reveals more than 90% homologous region between Rac1 and Rac2 proteins. A point mutation that leads to a dominant negative form of Rac2 (D57N) has been identified in infant patients characterized with recurrent bacterial infection and failure of wound healing resulting from defective neutrophil function (62–64). Although there is reduced T and B cell count in the patient, serum Ig level is normal except for one patient that had hypogammaglobulinemia (64). One of the patients harboring a homozygous mutation in Rac2 (W56X) that leads to a complete loss of the protein developed progressive B cell lymphopenia and hypogammaglobinemia (64). Based on studies of mice that lack Rac1 and Rac2, their function in multiple cellular processes, including proliferation, survival, adhesion, and migration have been implicated. In contrast to the B cell specific Rac1 knockout mice that do not present an obvious alteration of B cell functionality, Rac2 deficiency or combined deficiency of Rac1 and Rac2 (Rac1^B^Rac2^−/−^) leads to developmental block of B cells at the immature/transitional B cell stage. A study by Tybulewicz et al. shows that this is probably not due to a differentiation arrest of the transitional B cells, since ectopic expression of the anti-apoptotic gene Bcl-xl can partly rescue the differentiation defect of the Rac1^B^Rac2^−/−^ immature/transitional B cells. Instead, the defective migration toward chemokines is likely to be the reason why Rac1^B^Rac2^−/−^ B cells are unable to enter the white pulp where crucial survival signals to the mature B cells are available. This leads to a large reduction of the mature B cell population in the spleen including marginal zone B cells and follicular B cells (65). Defective entry of mature B cells into the white pulp makes it difficult to study the role of Rac1 and Rac2 in antigen-activated B cells. To circumvent this issue, Rac proteins were inducibly deleted by Tamoxifen in the mature B cell population (Rac1^B−ERT2^Rac2^−/−^) (66). The TI response to TNP-LPS of Rac1^B−ERT2^Rac2^−/−^ B cells is greatly compromised, with reduced level of antigen specific IgM and IgG3, whereas the TD response to TNP-SRBC in these mice seems comparable to wildtype mice, with a normal GC response and plasma cell output. Notably, Rac1^B−ERT2^Rac2^−/−^ mice have increased serum titer of antigen specific IgG2b. *In vitro* analysis of Ig class switching reveals that the Rac1^B−ERT2^Rac2^−/−^ B cells have increased capacity to switch to IgG2b, possibly attributed to increased gamma2b germline transcript. In addition, B cell activation induced by BCR cross-linking is compromised in Rac1^B−ERT2^Rac2^−/−^ B cells and associated with reduced cell proliferation and survival. This could be caused by compromised BCR signaling and upregulation of BAFF-R.

### CIP4

CIP4 (Cdc42 interacting protein 4) belongs to the Fes–CIP4 homology-Bin/Amphyphysin/Rvsp (F-BAR) family of proteins, which includes FBP17 (formin binding protein 17), and Toca-1 (transducer of Cdc42-dependent actin assembly 1). CIP4 interacts with Cdc42 and is a downstream target of activated GTP-bound Cdc42 (54). Similar to mice with Cdc42-deficient B cells, mice completely devoid of CIP4 have normal B and T cell development but reduced germinal center formation and decreased production of high affinity IgG in response to NP-KLH (67). Since CIP4 was deleted in all cells, the specific role of CIP4 in GC B cells and T cells was not examined. CIP4-deficient T cells had decreased migration and integrin-mediated adhesion under sheer forces, suggesting a defect in entry of Tfh cells into the GC.

### TC10/RhoG

TC10/RhoG is an atypical Rho GTPase identified as a member of the ras homolog gene family (68). TC10/RhoG is a member of the Rho family of GTPases that shares 72–62% sequence identity with Rac1 and Cdc42, respectively (69). In contrast to the marked defect of Cdc42-deficient B cells, specific deletion of TC10 had little effect on B cell development or differentiation into GC B cells, indicating that Cdc42 may compensate for loss of TC10 (70). Indeed, deletion of both Cdc42 and TC10 in B cells led to much reduced B cell proliferation in response to LPS and CpG stimulation.

## WASp Family of Actin Regulators

The Rho GTPases activate the Wiskott-Aldrich syndrome protein (WASp) family of actin regulators. The WASp family of proteins includes WASp, neuronal (N)-WASp, and WASp-family verprolin-homologous protein (WAVE)/suppressor of the cyclic AMP receptor (SCAR) 1–3, WASp and SCAR homolog (WASH), and junction-mediating and regulatory protein (JMY) (71–73). WASp family proteins are characterized by high homology in the C-terminal domain consisting of the verprolin cofilin acidic (VCA) domain though which they can bind to globular actin and the Arp2/3 complex. The N-terminus of the protein show higher variability likely linked to cell-specific functions. At rest, WASp and N-WASp resides in an auto-inhibited conformation due to an intramolecular interaction between the VCA domain and the GTPase-binding domain (74–76). Upon binding of Cdc42, the auto-inhibited conformation is released and exposes the VCA domain that allows for recruitment of the Arp2/3 complex and actin polymerization. Rac1 and Rac2 regulate activation of the multimeric WAVE/Scar regulatory complex to stimulate actin polymerization by the VCA domain (77–79). WASp was the first identified member due to that its loss-of-function leads to the severe immunodeficiency disease Wiskott-Aldrich syndrome (WAS), initially described by Alfred Wiskott in 1937 and Robert Aldrich in 1954 (Wiskott A, Familiärer, angeborener Morbus Werlhofii? Monatsschr Kinderheilkd 1937; 68:212-216; Aldrich RA, Pediatrics 1954; 13:133–139).

### WASp and N-WASp

WASp is uniquely expressed in hematopoietic lineage cells whereas N-WASp that shares 50% homology with WASp in the amino acid sequence is ubiquitously expressed. Humoral immunodeficiency caused by mutations in the *WAS* gene encoding WASp is associated with failure to respond to common pathogens and up to 40–70% of patients developing autoimmune disease with high titers of autoantibodies (80–85).

WAS patients have normal to slightly reduced absolute numbers of circulating B cells, however, have reduced MZ B cells and dysmorphic GC in spleen (80, 86). Although the proportion of memory B cells remains intact, WAS patient memory B cells have reduced responsiveness to BCR activation probably due to impaired BCR signaling (87). WASp^−/−^ mice have normal B cell development and FO B cells, but reduced number of MZ B cells and MZ precursor T2-MZP cells (88–90). This leads to reduced capacity to respond to TI antigens TNP-Ficoll and TNP-dextran, likely due to a combined effect of reduced number of MZ B cells and decreased antigen delivery by the MZ B cells to the B cell follicle (88, 90, 91). WASp^−/−^ mice have slightly reduced capacity to form high affinity IgG antibodies to TD antigen NP-KLH and particulate antigen SRBCs (88, 90–92). WASp^−/−^ B cells have decreased formation of the immune synapse upon BCR activation *in vitro* (89, 93) and reduced capacity to from long membrane extensions (49). Despite this defects in the BCR response, WASp^−/−^ B cells can present antigen and induce T cell activation similar to wildtype B cells, at least *in vitro* (94, 95). WASp acts as a negative regulator for autoreactive B cells since both WAS patients and WASp^−/−^ mice develop broad range IgM and IgG autoantibodies associated in mice with spontaneous generation of GCs (81, 85, 95). Moreover, WASp^−/−^ B cells are hyper responsive to B cell receptor and Toll-like receptor (TLR) signals *in vitro*, thereby promoting a B cell–intrinsic break in tolerance. To understand the B cell intrinsic defects, WASp^flox/flox^ mice were bred mb1-Cre mice to delete WASp specifically in B cells. These WASp^B^ mice have high titers of autoreactive IgM and IgG and form large GCs in the absence of antigen challenge (91, 96). To reveal the unique and redundant role of WASp and N-WASp in the GC response, WASp^−/−^ mice or WASp^flox/flox^ mice were bred to N-WASp^flox/flox^ mice and CD19-Cre or mb1-Cre to delete WASp and N-WASp specifically in B cells. Analysis of WASp^−/−^N-WASp^B^ and WASp^B^N-WASp^B^ mice revealed a reduced response to NP-KLH with small GCs that lost LZ and DZ integrity and failure to generated high affinity NP-specific IgG antibodies (95, 97). Strikingly, N-WASp deletion in WASp^−/−^ B cells lowered the autoreactive antibodies and GCs, suggesting that N-WASp deletion protects mice from developing autoimmune disease (95, 97). Interestingly, N-WASp-deleted B cells (that express normal WASp) have increased BCR synapse response associated with development of autoantibodies in N-WASp^B^ mice (93). This indicates that WASp and N-WASp serve both unique and redundant roles in BCR signaling to B cell activation. WASp-deficient follicular T (Tfh) cells show defective activation and proliferation and is likely to contribute to altered antibody production in WAS patients and WASp^−/−^ mice (98). Moreover, WASp deficiency in regulatory B cells leads to exacerbated experimental autoimmune arthritis (99).

The *WAS* gene is localized on the X chromosome and only boys are affected by *WAS* mutations. Studies of asymptomatic female WAS carriers has revealed that while haematopoietic stem cells have largely random X chromosome inactivation, there is a strong selective advantage for B and T cells that express WASp during development and differentiation (88, 89). By analysis of WASp^+/−^ heterozygous mice and WT:WASp^−/−^ bone marrow chimeric mice, a strong advantage was detected for WASp-expressing FO B cells and MZ B cells in the spleen, as well as GC B cells in Peyer's patches (88, 89). It was later shown that WASp^−/−^ B cells competed equally well with wildtype B cells among GC B cells, both DZ and LZ cells, whereas WASp^−/−^N-WASp^B^ had selective disadvantage in contribution to the GC B cells (95). This suggests that WASp together with N-WASp are needed for a normal GC response to prevent selection of autoreactive B cells. Gene therapy for WAS patients is currently evaluated in several centers and has shown success and ameliorate the autoreactive B cells. Gene therapy may provide a future curative option alongside haematopoietic stem cell transplantation (100).

### WASp-interacting Protein (WIP)

WIP was originally cloned as a WASp interacting protein using a yeast two-hybrid system. WIP interacts with the N-terminal WASp homology domain (WH) 1 domain of both WASp and N-WASp and is essential for their stability (101–103). Three pedigrees of WIP deficient patients have been reported so far (104–106). Their symptoms highly resemble those of WAS patients, however, with milder thrombocytopenia and earlier onset of severe infections and T cell deficiency (107). Similar to WAS patients, WIP deficient patients have elevated serum IgE titer and normal to elevated IgG and IgM antibody titer, suggesting abnormal B cell responses (105, 107). WIP^−/−^ B cells show reduced B cell homing, chemotaxis, survival, and differentiation due to an altered CD19 cell surface dynamics (108). Upon NP-KLH immunization, WIP^−/−^ mice failed to form GCs and have reduced NP-specific antibody responses. This was caused by reduced activation of phosphatidylinositol-4,5-bisphosphate 3-kinase (PI3K) in WIP^−/−^ B cells. WIP has important function in B cells, independent of its binding to WASp, by direct binding to actin (109). B cells expressing WIP lacking the actin binding domain (ABD) of WIP (WIPΔABD) have reduced BCR induced actin foci and reduced signaling with PI3K to p-Akt. Using NP-KLH immunization of WT:WIP^−/−^ mixed bone marrow chimeras, WIP^−/−^ B cells are less efficient at differentiating into GC B cells in a competitive environment. However, in a non-competitive setting, GC responses are comparable to WT mice but WIP^−/−^ mice are impaired in producing high-affinity antibodies (109). It was recently shown in T cells that WIP bridges Dock8 to WASp and actin and that Dock8 GEF activity is essential for TCR-driven WASp activation and F-actin assembly (110). It is plausible that WIP serves a similar function in BCR signaling.

## Conclusions and Future Perspectives

Positive selection of B cells in GCs depends on the BCR affinity and requires help from Tfh cells. Selected B cells have three possible fates: to become a plasma cell, a memory cell, or to re-enter the DZ for more rounds of mutation and selection. Absolute high affinity is suggested to drive GC B cells to differentiate into plasma cells, whereas relatively lower affinity lead to differentiation into memory B cells. However, several questions remain elusive about how variable BCR affinity is discriminated and how cell fate decisions within the GCs are regulated. Some recent studies suggests that the actin regulators are involved in the antigen retrieval of GC B cells from FDC by polarization of the lysosomes to the BCR-antigen immune complex and by generating mechanic forces. This raises the interesting question of whether dysregulated actin dynamics can directly influence the fate decision of GC B cells and eventually impact on the quality and efficacy of humoral immune responses.

**Figure 2:**
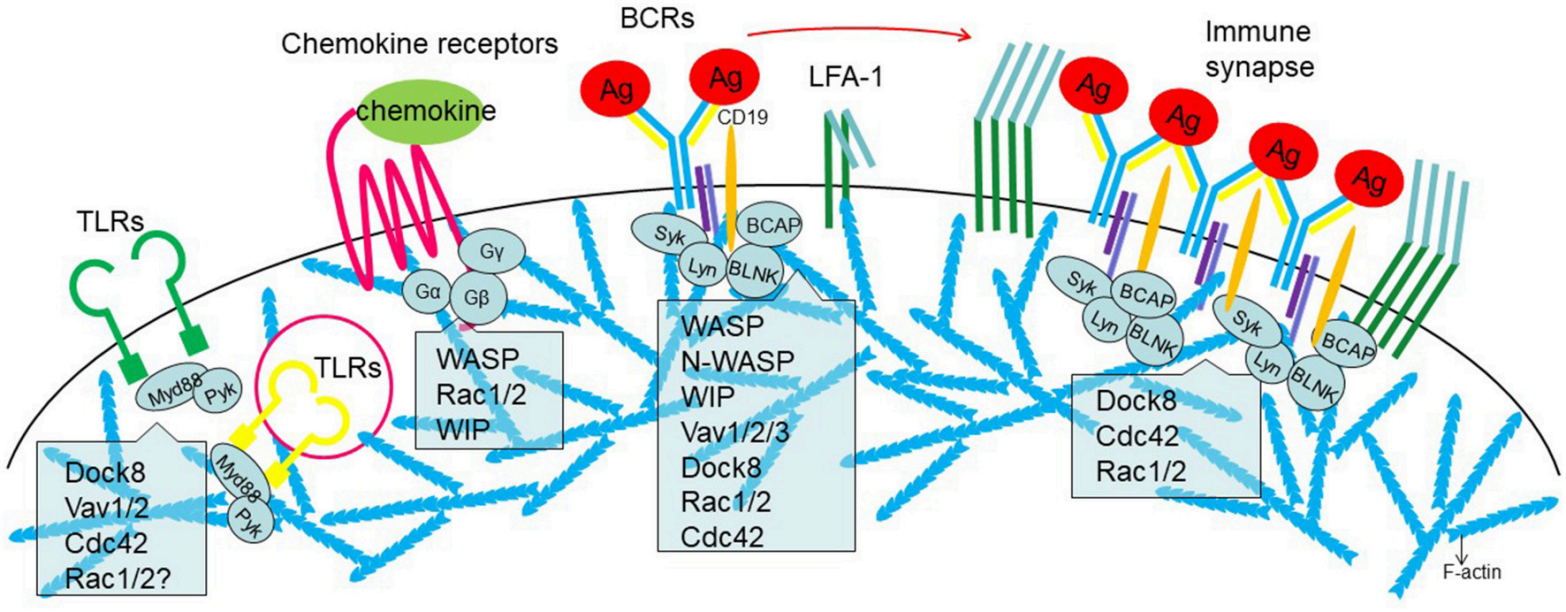
Involvement of actin regulators in the receptor signaling during B cell activation. From Left to right, surface or cytosolic Toll-like receptors (TLRs), chemokine receptors, B cell receptors (BCRs), and immune synapse. Role of actin regulators discussed is indicated.

Deficiency in cytoskeletal regulation often influences the cell fate decision to become a FO B cell or MZ B cells. Mice devoid of Dock8, Cdc42, Rac2, WASp, WASp plus N-WASp, and WIP have reduced number of MZ B cells. Reduced MZ B cells may lead to decreased delivery of antigens to the FDC network, as is the case for mice lacking WASp and WASp plus N-WASp in B cells (88, 92). The reduction in MZ B cells may be related to changes in BCR signaling strength. Data support that the strength of BCR signaling in the transitional B cells that enter secondary lymphoid organs is important. MZ B cells are favored by low BCR signaling whereas FO B cells depend on high BCR signaling (111). Within the GC, BCR signaling may be of less importance and BCR as an endocytic receptor for antigen capture, processing, and presentation may be more important during affinity maturation (6). In contrast with naive and memory B cells, which extract antigen in the synapse center, GC B cells extract antigen using several small peripheral clusters. Both naive and GC B cell synapses require proximal BCR signaling, but GC B cells signal less through the protein kinase Cβ-NFκB allowing them to more stringently regulate antigen binding (10). A unifying conclusion from the studies discussed here is that there is enormous redundancy in signaling pathways leading to Arp2/3 mediated actin polymerization (Figure 2). However, approaches to use double-deficiency of two potential redundant factor such as WASp and N-WASp have led to surprising results. This is likely due to that the signaling threshold for BCR activation is fine-tuned to achieve a balance between antigen affinity and antigen extraction to avoid differentiation of autoreactive B cells and malignant B cell clones.

## Author Contributions

Both authors listed have made a substantial, direct and intellectual contribution to the work, and approved it for publication.

### Conflict of Interest Statement

The authors declare that the research was conducted in the absence of any commercial or financial relationships that could be construed as a potential conflict of interest.
